# Cost effectivenes of erlotinib versus chemotherapy for first-line treatment of non small cell lung cancer (NSCLC) in fit elderly patients participating in a prospective phase 2 study (GFPC 0504)

**DOI:** 10.1186/1471-2407-12-301

**Published:** 2012-07-20

**Authors:** Chouaid Christos, Le Caer Hervé, Locher Chrystelle, Dujon Cecile, Thomas Pascal, Auliac Jean Bernard, Monnet Isabelle, Vergnenegre Alain

**Affiliations:** 1 AP-HP Hôpital St-Antoine, AP-HP, UMPC, Paris, France; 2 CH de Draguignan, Draguignan, France; 3 CH de Meaux, Meaux, France; 4 CHI de Versailles, Versailles, France; 5 CH de Gap, Paris, France; 6 CH de Mantes La Jolie, Mantes La Jolie, France; 7 CHI de Creteil, Creteil, France; 8 CHU de Limoges, Limoges, France; 9 Service de Pneumologie, Hôpital Saint-Antoine, 84 rue du boulevard Saint-Antoine, 75012, Paris, France

**Keywords:** Cost-utility, Erlotinib, Non-small cell lung cancer, Elderly patients, Phase II trial

## Abstract

**Background:**

The median age of newly diagnosed patients with non-small cell lung cancer (NSCLC) is 67 years, and one-third of patients are older than 75 years. Elderly patients are more vulnerable to the adverse effects of chemotherapy, and targeted therapy might thus be a relevant alternative. The objective of this study was to assess the cost-effectiveness of erlotinib followed by chemotherapy after progression, compared to the reverse strategy, in fit elderly patients with advanced NSCLC participating in a prospective randomized phase 2 trial (GFPC0504).

**Methods:**

Outcomes (PFS and overall survival) and costs (limited to direct medical costs, from the third-party payer perspective) were prospectively collected until second progression. Costs after progression and health utilities (based on disease states and grade 3–4 toxicities) were derived from the literature.

**Results:**

Median overall survival, QALY and total costs for the erlotinib-first strategy were respectively 7.1 months, 0.51 and 27 734 €, compared to 9.4 months, 0.52 and 31 688 € for the chemotherapy-first strategy. The Monte Carlo simulation demonstrates that the two strategies do not differ statistically.

**Conclusion:**

In terms of cost effectiveness, in fit elderly patients with NSCLC, erlotinib followed by chemotherapy compares well with the reverse strategy.

## Background

The US National Institutes of Health estimated that $89 billion was spent on cancer care in the United States in 2007, and that the total economic burden reached $219.2 billion when indirect costs associated with lost productivity and death were taken into account. Recent trends suggest that the growth in cancer spending will accelerate, owing to costly new treatments and the increasing number of cancer patients. These costs may increase further still with the introduction of novel targeted therapies [1]. Lung cancer is the second most common malignancy in the US and the leading cause of cancer-related death. Non small-cell lung cancer (NSCLC) accounts for 85% of lung cancers, and most patients already have advanced or metastatic disease at diagnosis [2]. Between 30% and 40% of NSCLC cases are diagnosed in patients over 70 years of age, raising specific issues of age, comorbidity and toxicity [3]. Most elderly patients are either under-treated or receive non validated schedules [3,4]. They are also largely under-represented in therapeutic trials, and little clinical research takes their specificities into account [4].T The value of specific studies of elderly subjects has been clearly demonstrated [4]. The recommended first-line treatment for patients under 65 with metastatic NSCLC and good performance status consists of dual-agent platinum-based chemotherapy. There is no consensus on the management of elderly NSCLC patients, although adapted platinum-based chemotherapy seems feasible in high selected subjects [4-6]. Single-agent and dual-agent therapy without a platinum salt also seems possible for patients selected on the basis of a geriatric assessment taking comorbidities into account. Among the available non platinum-based chemotherapy regimens, the docetaxel-gemcitabine (DG) combination is considered one of the most promising [7-9]. Targeted therapies have given promising results in elderly populations. In the pivotal BR21 study, second-line erlotinib had the same efficacy in the subgroup of patients over 70 years old as in the entire population [10]. Targeted therapies are also a potential first-line option for elderly patients with advanced NSCLC [11]. Indeed, erlotinib was well tolerated in this population and there was a significant improvement in key symptoms [11]. One difficulty in this setting is the heterogeneity of elderly populations. The use of a co-morbidity score and a comprehensive geriatric assessment (CGA) can identify a more homogenous group of fit elderly patients [9]. The objective of this cost analysis was to assess the cost-effectiveness of first-line erlotinib followed by chemotherapy after progression, compared to the reverse strategy, in fit elderly patients with advanced NSCLC participating in the GFPC0504 study, a randomized phase 2 trial [12].

### Patients and methods

#### Study design and population

The GFPC 0504 study was a multicenter, open-label, randomized phase II trial. It involved patients with previously untreated stage IIIB or IV NSCLC. It compared erlotinib followed, after progression, by weekly chemotherapy (docetaxel 30 mg/m² for 6 consecutive weeks and gemcitabine 900 mg/m2 at weeks 1, 2, 4 and 5, followed by a two-week treatment-free period) (Arm A) to the reverse strategy (arm B). The primary endpoint was second-progression-free survival. Overall survival was a secondary endpoint.

#### Costs

Costs were estimated from perspective the French health care system, from randomization until death. All resources consumed during the first and second treatment lines were prospectively collected on a per patient basis. Resources consumed were comprised of chemotherapy drugs, erlotinib, supportive treatments (including recombinant human erythropoietin, antiemetics, colony-stimulating factors, antibiotics, management of adverse effects, etc.), transfusion, and hospitalization for any reason. The specific unit costs are reported in Table 1[13,14]. Costs incurred after the second disease progression period were derived from a representative French nationwide sample of 428 patients, using chart review to assess the mean direct monthly cost of NSCLC patient management after the second line progression [15]. Specifically, the costs included outpatient and inpatient services, care provision at skilled nursing facilities, outpatient and inpatient drugs and other medications, nursing care organization, home health visits (including medications), and durable medical equipment. Assuming a yearly increment of 3.5%, one month of palliative care cost 2324 euros (2011 value).

**Table 1:** Model inputs

	**Estimates**	**Low**	**High**	**Source**
**Health state utilities**	0.673	0.27	0.80	[16,17]
Stable disease on oral therapy	0.653	0.26	0.78	[16,17]
Stable disease on IV therapy	0.473	0.19	0.56	[16,17]
Progressive disease	0			[16,17]
Death				
**Cost of medical services and drugs (€)**	2174.7	1627	3021	[12,13]
Erlotinib 30 days supply (150 mg)	9.1/mg			[12,13]
Docetaxel	0.2/mg			[12,13]
Gemcitabine	368			[12,13]
Hospitalization at home (day)	422			[12,13]
Day-ward hospital	557.40			[12,13]
G-CSF injection (per cycle)	220.53			[12,13]
Erythropoietin (per injection)	2324			[15]
Palliative care after progression (per month)				

#### Utilities

Utilities were derived from UK community population-based studies in advanced NSCLC [16,17], which used the standard gamble interview and visual analog scales to assess quality of life (Table 1).

#### Cost-utility analysis

The incremental cost-effectiveness ratios (ICER) were calculated. These ratios correspond to the difference in costs divided by the difference in effectiveness (expressed in QALY) between two strategies.

#### Statistical analysis

Second-progression-free survival was calculated from randomization to disease progression (after the second line of treatment if the patients received 2 lines, after the first line if the patient progressed and did not receive a second line), or death of any cause, or the last on-trial tumor assessment. OS was calculated from randomization to death from any cause, or the last date the patient was known to be alive. PFS and OS were assessed by the Kaplan-Meier method. Statistical analyses used SAS software version 9.01 (Institute INC, Carry, USA).

#### Assessing uncertainty

The uncertainty was evaluated by using one-way sensitivity analysis, sequentially varying the estimates for a given model parameter while keeping the other parameters constant, within a range of likely values derived from confidence intervals or reasonable ranges as determined from published sources. In addition, multivariate probabilistic sensitivity analysis was performed using second-order Monte Carlo simulation, in which the model inputs (time to second progression, OS, utilities and costs) were drawn from individual data. Specific distributions were assigned to utility data by using published means and standard deviations to specify the normal distribution. A simulation with 10 000 replications of the model was then used to obtain the 95% non-parametric confidence intervals for the costs and effectiveness parameters, and to determine the proportion of replications in each quadrant of the cost-effectiveness plane. The multiway sensitivity analysis was presented in radar screen format, in which the X-axis shows the difference in effectiveness and the Y-axis the difference in costs between two strategies. The 10 000 replications are represented by dots.

## Results

**Figure 1:**
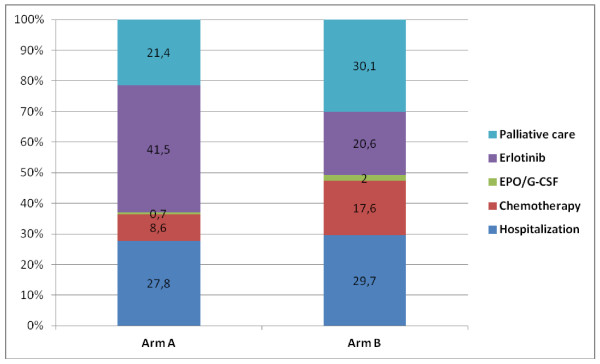
Per patient mean cost in Arm A (erlotinib followed by docetaxel and gemcitabine (DG) and Arm B (DG followed by erlotinib).

**Figure 2:**
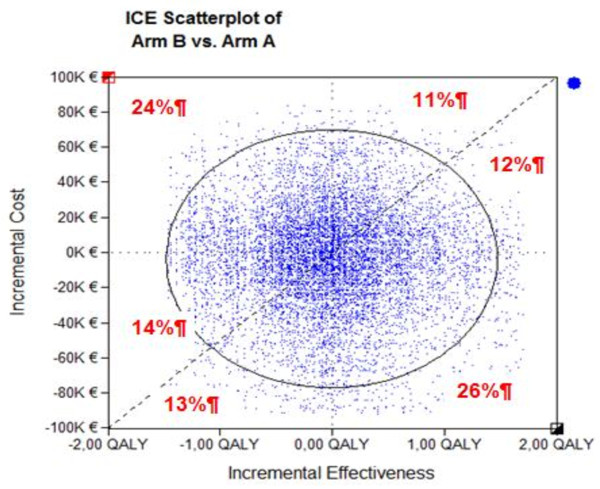
**Multivariate probabilistic sensitivity analysis (rresult of 10 000 replications). **Arm A: erlotinib followed by docetaxel and gemcitabine (DG); Arm B: DG followed by erlotinib (ICE: incremental cost effectiveness).

Between July 2006 and November 2008, 22 centers enrolled 100 patients in this study. Demographic variables measured int the two groups did not differ in a statistically way (Table 2). As already reported, there was no significant difference in the Charlson scores, co-morbidities, or geriatric assessment scores [12]. There was no significant difference between the two arms in terms of the time to second progression (5.8 and 7.5 months respectively in arms A and B, p = 0.53) or median OS (7.1 and 9.4 months, p = 0.26); QALY values were respectively 0.51 ± 0.44 and 0.52 ± 0.41 and costs were 27 734 ± 19801 and 31 688 ± 22693 €. The distribution of these costs differed between the two arms: palliative care represented respectively 21.4% and 30.0% of total costs; chemotherapy 8.6% and 17.6%, and erlotinib 41.5% and 19.7%, in arms A and B (Figure 1). The Monte Carlo simulation demonstrates that the two strategies do not differ statistically. Multivariate probabilistic sensitivity analysis (results of 10 000 replications) showed that the two strategies had equivalent cost-effectiveness (Figure 2), as confirmed by varying the utility values and the cost of palliative care (Table 3).

**Table 2:** Patients characteristics: Arm A: erlotinib followed by docetaxel plus gemcitabine (DG) after progression; Arm B: DG followed by erlotinib after progression (* no significant difference, ** p = 0.013**)**

	**Arm A**	**Arm B**
	**n = 48**	**n = 51**
**Age** mean (years)	76 ± 5	76 ± 4*
**Gender** male (%)	29 (60 %)	30 (59 %)*
**Smoker**	6 (13 %)	8 (16 %)*
Current	26 (54 %)	25 (49 %)
Former	15 (31 %)	15 (29 %)
Never smoker	1 (2 %)	3 (6 %)
Unknown		
**Performance status**	22 (47 %)	21 (41 %)*
0	21 (45 %)	28 (55 %)
1	4 (9 %)	2 (4 %)
2		
**Stage**	6 (13 %)	4 (8 %)*
IIIB	42 (87 %)	47 (92 %)
IV		
**Histology**	11 (23 %)	8 (16 %)**
Squamous cell	28 (58 %)	29 (57 %)
Adenocarcinoma	9 (19 %)	14 (28 %)
Undifferentiated		
Second line treatment	29 (60 %)	24 (47 %)**

**Table 3:** **One way sensitivity analysis**

	**Arm A**	**Arm B**	**ICER B/A**
	**Costs** € **/ utility**	**Costs** €**/ utility**	€**/QALY**
**Base case**	27 734 €/0.51	31 688 €/ 0.52	395400
**Utility of patients treated by erlotinib**			
−20% (0.538)	27 734 €/ 0.48	31 688 €/ 0.51	131800
−10% (0.606)	27 734 €/0.51	31 688 €/ 0.52	395400
+10% (0.740)	27 734 €/ 0.54	31 688 €/ 0.54	NA
+20% (0.807)	27 734 €/ 0.57	31 688 €/ 0.55	NA
**Post-progression cost**			
1627 €	25 954 €/ 0.51	28 787 €/ 0.52	283300
3021 €	29 514 €/ 0.51	34 588 €/ 0.52	507400
**Erlotinib tariff**			
−30%	24 282 €/ 0.51	29 788 €/ 0.52	550600
+ 30%	31 186 €/ 0.51	33 588 €/ 0.52	240200

Arm A: erlotinib followed by docetaxel and gemcitabine (DG); Arm B: DG followed by erlotinib.

## Discussion

This analysis showed no significant difference in patient outcomes between first-line erlotinib followed by chemotherapy after progression and the reverse sequence. However, the erlotinib-first strategy was less costly, and the ICER of the chemotherapy-first strategy relative to the erlotinib-first strategy was 395 400 € per QALY. These results were obtained in a highly specific population of fit elderly patients selected with a CGA. The main originality of this study is that the second-line treatment was fixed in each arm, thus allowing us to evaluate the performance of the entire treatment strategies. In advanced NSCLC, economic analyses are usually limited to either first- or second-line treatment. Sand studies of doublets without platinum salts are rare in the first-line setting [2].

A study done in Greece [18] compared the docetaxel/gemcitabin combination with docetaxel monotherapy in untreated patients with advanced NSCLC. It showed an incremental cost per life-year gained (LYG) of 9538 euros with the combination. The probability of being cost-effective was 91% at a threshold of 20 000 euros, 97% at 35 000 euros and 98% at 50 000 euros.

In the second-line setting, the ICER of erlotinib versus placebo [19] was explored by using resource utilization determined from individual patient data in the BR.21 trial database (a pivotal trial in this setting). The trial involved 731 patients (488 in the erlotinib arm and 243 in the placebo arm). The ICER of erlotinib was $94 638 per LYG (95% confidence interval = $52 359 to $429 148). The major drivers of cost-effectiveness included the magnitude of the survival benefit and the cost of erlotinib. Subgroup analyses suggested that erlotinib might be more cost-effective in never-smokers. There was no specific analysis of elderly patients included in this study.

The cost-effectiveness of erlotinib has also been compared with that of other agents (docetaxel and pemetrexed) licensed for second-line treatment of advanced NSCLC [20]. In a model-based analysis, second-line treatment with erlotinib, docetaxel and pemetrexed yielded respectively 0.42, 0.41, and 0.41 QALY, and total costs were US$ 37 000, 39 100 and 43 800. Again, there was no specific analysis of elderly patients. A more recent cost-utility analysis compared erlotinib and docetaxel for second-line management of advanced NSCLC within the UK National Health Service. The authors used a health-state transition model, based on the two pivotal phase III studies of erlotinib versus best supportive care and docetaxel versus best supportive care, to estimate direct costs, QALY, and the subsequent net monetary benefit. Erlotinib was associated with lower total costs (£13 730 versus £13 956) and a gain in QALY [16]. In a recently, retrospective real world cost-effectiveness study, on second line setting, erlotinib and docetaxel are statistically equivalent in terms of treatment costs and overall survival [21].

Regarding the burden of NSCLC in terms of health-related quality of life, little information is available on the preferences of patients or society with respect to disease states [22,23]. We used data from Nafees et al. [17], who adapted existing health-state descriptions in metastatic breast cancer to evaluate the utilities of patients receiving second-line treatment for NSCLC. Each health state describes the symptom burden of a disease and its functional impact. More recently, Lewis [16] used the same method to establish health utilities for erlotinib therapy, based on data for 154 members of the UK general population, using the euroQol EQ-5D instrument. We used the results of both studies to test the robustness of our model with varying utility values.

One advantage of our study is the prospective collection of cost data, at least until second progression. In contrast, management costs after the end of active treatments were derived from a 2004 national database. In addition, our analysis was limited to direct lung cancer-related medical costs: indirect costs such as lost productivity and caregiver salaries were not included. Also, the way in which we expressed utilities reflects the value from the point of view of society rather than that of the patients concerned. Finally, it is uncertain whether these utilities are fully relevant to our population of elderly patients. However, our sensitivity analyses compensated for these limitations, as the conclusions based on the base-case scenario were unaffected when we varied the different model parameters. One limitation of our analysis is that EGFR patients status was not knew and we a unable to analyze the ICER of erlotinib first line treatment in the subgroup of patient with a EGFR mutation.

## Conclusion

In terms of cost-effectiveness, in fit elderly patients with NSCLC, erlotinib followed by chemotherapy compares well with the reverse strategy.

## Competing interest

C. Chouaid has received consultancy fees (less than 10 000 USD) from Roche Pharmaceuticals, Amgen, GSK, Astra Zeneca and Lilly. A. Vergnenegre has received consultancy fees (less than 10 000 USD) from Roche Pharmaceuticals, Astra Zeneca and Lilly. The other authors declare that they have no conflicts of interest.

## Author contributions

C. Chouaid had full control of the study design, data analysis and interpretation, and manuscript preparation. CC, LH and VA conceived and performed the statistical analysis, LC, DC, TP and IM particped in the design and ccordination and helped to draft the manuscript. The final draft manuscript was approved by all the authors.

## The GFPC0504 team

Pr. Vergnenegre A, Pr. Melloni B, CHU Limoges;

Pr Prof Barlesi F, Dr Greillier L, CHU Marseille;

Dr LeCaer H, Dr Barriere JR, CH Draguignan;

Dr Gerinière L, CHU Lyon;

Dr Bombaron P, CH Mulhouse;

Dr Crequit J, CH Beauvais;

Dr Auliac JB CH Mantes;

Dr Le Treut J, CH Aix;

Dr Berard H, HIA Toulon;

Dr Thomas P, Dr Muller P, CH Gap;

Dr Fournel P CHU St Etienne;

Dr Robinet G, Dr Andre M, CHU Brest;

Dr Grivaux M, Dr Locher C CH Meaux;

Dr .Berdah JF Clinique Esperance Hyeres;

Pr. Chouaid C, Dr Baud M CHU Paris St Antoine;

Dr Bota S, CHU Rouen CN;

Dr Paillotin D CHU Rouen BG;

Dr Corre R, Dr Lena H CHU Rennes;

Dr Chouabe S, CH Charleville;

Dr Delhoume JY CH Perigueux;

Dr Dujon C CH Le Chesnay;

Dr Jullian H, Dr .Simonian H, CH Martigues;

Dr David P, CH Elbeuf;

Dr Falchero L, CH Villefranche;

Dr Janicot H, CHU Clermont-Ferrand;

Dr Monnet I, CHI Creteil

## Pre-publication history

The pre-publication history for this paper can be accessed here:

http://www.biomedcentral.com/1471-2407/12/301/prepub
